# Supplementary material for: Conditional chemoconnectomics (cCCTomics) as a strategy for efficient and conditional targeting of chemical transmission
Source: eLife. 2024 Apr 30;12:RP91927. doi: 10.7554/eLife.91927 (PMC11060718; doi:10.7554/eLife.91927)
Supplement: Figure 3—source data 3. [file elife-91927-fig3-data3.zip › Figure 3 source data3 part 1.zip!/Figure 3-Source data 3-part1/1063f3-1-CRISPResso_Report_kboyr13q.zip!/CRISPResso_on_1063f3_2/9.Alleles_frequency_table_around_sgRNA_aatcacaccagtgggagcac.pdf]

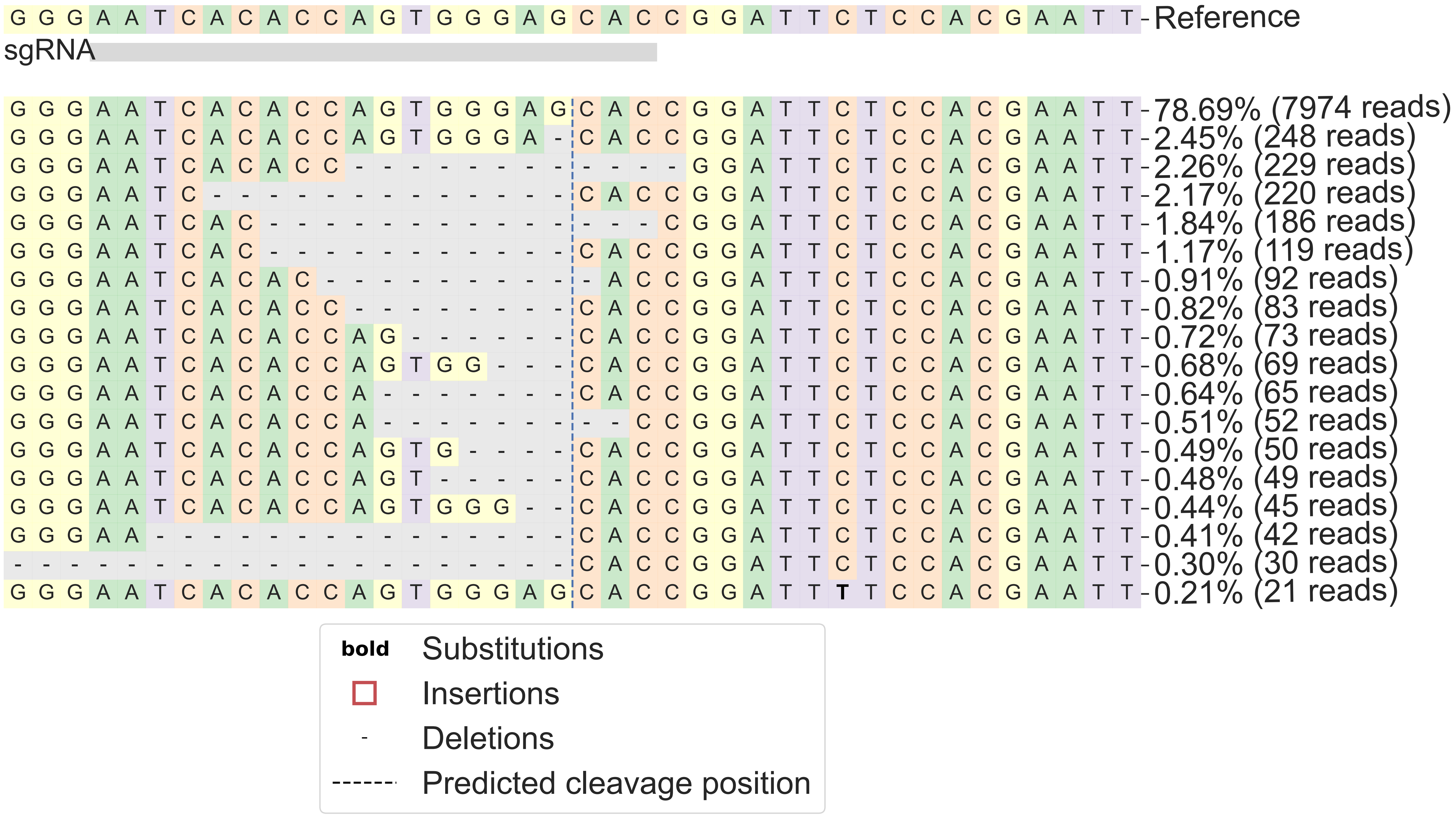
GGGAATCACACCAGTGGGAGCACCGGATTCTCCACGAATT Reference
sgRNA
GGGAATCACACCAGTGGGAGCACCGGATTCTCCACGAATT 78.69% (7974 reads)
GGGAATCACACCAGTGGGA-CACCGGATTCTCCACGAATT 2.45% (248 reads)
GGGAATCACACC------------GGATTCTCCACGAATT 2.26% (229 reads)
GGGAATC-------------CACCGGATTCTCCACGAATT 2.17% (220 reads)
GGGAATCAC--------------CGGATTCTCCACGAATT 1.84% (186 reads)
GGGAATCAC-----------CACCGGATTCTCCACGAATT 1.17% (119 reads)
GGGAATCACAC----------ACCGGATTCTCCACGAATT 0.91% (92 reads)
GGGAATCACACC--------CACCGGATTCTCCACGAATT 0.82% (83 reads)
GGGAATCACACCAG------CACCGGATTCTCCACGAATT 0.72% (73 reads)
GGGAATCACACCAGTGG---CACCGGATTCTCCACGAATT 0.68% (69 reads)
GGGAATCACACCA-------CACCGGATTCTCCACGAATT 0.64% (65 reads)
GGGAATCACACCA---------CCGGATTCTCCACGAATT 0.51% (52 reads)
GGGAATCACACCAGTG----CACCGGATTCTCCACGAATT 0.49% (50 reads)
GGGAATCACACCAGT-----CACCGGATTCTCCACGAATT 0.48% (49 reads)
GGGAATCACACCAGTGGG--CACCGGATTCTCCACGAATT 0.44% (45 reads)
GGGAA---------------CACCGGATTCTCCACGAATT 0.41% (42 reads)
--------------------CACCGGATTCTCCACGAATT 0.30% (30 reads)
GGGAATCACACCAGTGGGAGCACCGGATT**T**TCCACGAATT 0.21% (21 reads)
bold Substitutions
Insertions
- Deletions
Predicted cleavage position
